# Right hemisphere structural adaptation and changing language skills years after left hemisphere stroke

**DOI:** 10.1093/brain/awx086

**Published:** 2017-04-24

**Authors:** Thomas M. H. Hope, Alex P. Leff, Susan Prejawa, Rachel Bruce, Zula Haigh, Louise Lim, Sue Ramsden, Marion Oberhuber, Philipp Ludersdorfer, Jenny Crinion, Mohamed L. Seghier, Cathy J. Price

**Affiliations:** 1 Wellcome Trust Centre for Neuroimaging, University College London, UK; 2 Institute of Cognitive Neuroscience, University College London, UK; 3 Department of Brain, Repair and Rehabilitation, Institute of Neurology, University College London, UK

**Keywords:** stroke, language, chronic aphasia, recovery, decline, MRI

## Abstract

Stroke survivors with acquired language deficits are commonly thought to reach a ‘plateau’ within a year of stroke onset, after which their residual language skills will remain stable. Nevertheless, there have been reports of patients who appear to recover over years. Here, we analysed longitudinal change in 28 left-hemisphere stroke patients, each more than a year post-stroke when first assessed—testing each patient’s spoken object naming skills and acquiring structural brain scans twice. Some of the patients appeared to improve over time while others declined; both directions of change were associated with, and predictable given, structural adaptation in the intact right hemisphere of the brain. Contrary to the prevailing view that these patients’ language skills are stable, these results imply that real change continues over years. The strongest brain–behaviour associations (the ‘peak clusters’) were in the anterior temporal lobe and the precentral gyrus. Using functional magnetic resonance imaging, we confirmed that both regions are actively involved when neurologically normal control subjects name visually presented objects, but neither appeared to be involved when the same participants used a finger press to make semantic association decisions on the same stimuli. This suggests that these regions serve word-retrieval or articulatory functions in the undamaged brain. We teased these interpretations apart by reference to change in other tasks. Consistent with the claim that the real change is occurring here, change in spoken object naming was correlated with change in two other similar tasks, spoken action naming and written object naming, each of which was independently associated with structural adaptation in similar (overlapping) right hemisphere regions. Change in written object naming, which requires word-retrieval but not articulation, was also significantly more correlated with both (i) change in spoken object naming; and (ii) structural adaptation in the two peak clusters, than was change in another task—auditory word repetition—which requires articulation but not word retrieval. This suggests that the changes in spoken object naming reflected variation at the level of word-retrieval processes. Surprisingly, given their qualitatively similar activation profiles, hypertrophy in the anterior temporal region was associated with improving behaviour, while hypertrophy in the precentral gyrus was associated with declining behaviour. We predict that either or both of these regions might be fruitful targets for neural stimulation studies (suppressing the precentral region and/or enhancing the anterior temporal region), aiming to encourage recovery or arrest decline even years after stroke occurs.

## Teaser

Language difficulties after stroke are commonly thought to stabilise within a year. Hope *et al*. report surprising evidence to the contrary, showing that the language skills of patients with post-stroke aphasia continue to change even years after stroke. The changes are associated with structural adaptation in the intact right hemisphere.

## Introduction

Language deficits (aphasia) are some of the most feared consequences of stroke (Lam and Wodchis, 2010). There are thought to be more than a million aphasic stroke survivors in the USA alone, with a further 80 000 new cases per year (Ellis *et al.*, 2010). While recovery from these deficits can occur quickly, it has long been assumed that aphasic patients plateau in that recovery within the first year post-stroke, after which their language skills and any residual deficits will remain stable (Culton, 1969; Sarno and Levita, 1971; Teasell *et al.*, 2012). On this view, language function is effectively locked down after the functional reorganization that occurs in the early months after stroke. To the extent that this is true, the prognosis for patients whose symptoms persist beyond that first year, is bleak, and as a consequence even the world’s most advanced health systems provide little ongoing care or treatment for aphasics in that chronic phase (Teasell *et al.*, 2012). But a growing evidence base suggests that chronic aphasics’ language skills might be rather more dynamic than previously thought.

Most of this more encouraging evidence comes from therapeutic intervention studies, using speech and language therapies (Pulvermüller *et al.*, 2001; Moss and Nicholas, 2006; Taub, 2012), neural stimulation (Turkeltaub *et al.*, 2012; de Aguiar *et al.*, 2015), or drugs (Bakheit, 2004; Berthier *et al.*, 2011), either singly or in combination. These studies increasingly demonstrate that change is possible in chronic aphasia, suggesting that long-term plasticity or repair is possible. However, although compelling, the treatment effects in these studies often diminish over time after the treatment itself comes to an end (Menke *et al.*, 2009; Meinzer *et al.*, 2014). Plausibly, therapy effects might diminish over time because short-term memory is never properly consolidated.

The other source of evidence for long-term change is both rarer and harder to reconcile with the presumption of stability: studies that report what appears to be ‘spontaneous’ change in chronic aphasia—spontaneous in the sense that change occurs irrespective of any particular intervention. Spontaneous change is an accepted feature of recovery in the first few months (the acute phase) post-stroke (Culton, 1969; Lomas and Kertesz, 1978), but is rarely associated with the chronic phase; nevertheless, there are reports that it happens. Almost all of these studies report purely behavioural effects—the observation of single patients (Smania *et al.*, 2010; Stark, 2010) or small samples (Kertesz and McCabe, 1977; Hanson *et al.*, 1985), whose language skills appear to continue to improve beyond the first year post-stroke, apparently without overt or consistent intervention. One recent study complements that behavioural analysis with the repeated application of a functional MRI language paradigm, associating significant behavioural recovery with enhanced functional activation in right hemisphere homologues of left hemisphere language regions (Elkana *et al.*, 2013).

The presumption of stability in chronic aphasia is both popular and seemingly well-supported (Saur *et al.*, 2006; Toschke *et al.*, 2010; Marchina *et al.*, 2011; Maas *et al.*, 2012; Fridriksson *et al.*, 2013; Kwakkel and Kollen, 2013; Butler *et al.*, 2014); behavioural observations alone could only reverse it if they were very consistent indeed. And while the functional imaging study (Elkana *et al.*, 2013) adds a more mechanistic level of analysis, the sample used in that study was both small (*n* = 7) and highly unusual, because all of the participants were children when their strokes occurred—expressly because of the hope that spontaneous change would be more likely in that younger participant group. There is every reason to doubt that the effects reported there will generalize to more typical populations of chronic aphasics. In what follows, we search for evidence of spontaneous change in a larger and more typical sample.

## Materials and methods

### Patient data

Our patient data were extracted from our PLORAS database (Seghier *et al.*, 2016), which associates stroke patients, tested over a broad range of times post-stroke, with demographic data, behavioural test scores from the Comprehensive Aphasia Test (CAT, Swinburn, 2004), and high resolution T_1_-weighted MRI brain scans. Patients are excluded from the PLORAS database when there is evidence they have other neurological conditions (e.g. dementia, multiple sclerosis), contraindications to MRI scanning, are unable to see or hear the stimuli required to assess their language abilities, or have insufficient comprehension of the purpose of the study to provide consent for their participation. All patients are invited for repeat assessments if they were classified as impaired on at least one of our language tasks at first assessment. Brain imaging is also repeated unless the patient has new contraindications to MRI, does not want to be scanned again, lives a long way from the lab or there are time restrictions for retesting. Every effort was made to minimize variability caused by the testing itself for example due to the time of day or the order of scanning and behavioural testing, but priority in every case was given to the patients’ own comfort and convenience.

Our inclusion criteria for the current study were right-handed stroke patients who had: (i) no evidence of secondary stroke events between assessments (74 patients); (ii) at least two structural brain scans that were all more than 1 year post-stroke (71 patients); (iii) different behavioural assessments conducted around the time of each brain scan (71 patients); (iv) English as their first language (61 patients); (v) lesions that were >1 cm^3^ in the left hemisphere and <1 cm^3^ in the right hemisphere (34 patients); and (v) were right-handed pre-stroke (28 patients). When patients met the above criteria and had more than two brain scans that were acquired more than a year after their stroke, we picked the time points that maximized the test–retest interval for that patient. There were four patients for whom one CAT assessment was separated by more than 2 months from their closest scan (Table 1), but because the time between CAT and brain scan was small compared to the time between brain scans, the inclusion or exclusion of these patients does not affect our findings or conclusions. Summary details of the 28 patients who met these criteria are as follows: 10 females; mean/standard deviation (SD) of age at stroke onset = 51.7/10.4 years; mean/SD of time post-stroke when assessed = (T1) 50.7/43.7 months; (T2) 80.4/53.5 months; (T2 − T1) 30.7/25.8 months (Table 1).

**Table 1:** Individual patient data

**Patient no.**	**Age at stroke (years)**	**Sex**	**Lesion volume (cm^3^)**	**Time post stroke (months)**		**Spoke object naming**		**Auditory word repetition**		**Written object naming**		**Spoken action naming**		**Days between scan and behaviour tests (months)**	
				**T1**	**T2**	**T1**	**T2**	**T1**	**T2**	**T1**	**T2**	**T1**	**T2**	**T1**	**T2**
PS0005	30	F	33	64	166	74	61	65	65	67	67	69	69	0	0
PS0019	41	M	116	199	222	64	62	57	60	55	60	59	56	0	0
PS0041	64	M	4	15	27	61	60	65	65	62	60	69	69	0	0
PS0043	43	M	2	56	76	74	74	65	60	67	67	69	69	0	0
PS0069	66	M	76	17	67	43	45	–	–	55	67	39	39	0	−1
PS0082	51	F	35	16	54	60	61	51	50	62	67	63	59	0	0
PS0088	60	M	44	51	89	51	52	51	53	58	67	59	54	−9	0
PS0163	46	F	31	15	35	64	70	49	52	62	67	59	63	0	0
PS0171	58	M	115	54	59	53	60	50	51	58	67	47	69	0	0
PS0180	68	M	26	19	36	64	59	46	47	55	67	59	69	0	0
PS0184	63	M	65	52	56	50	51	57	48	47	51	39	49	0	0
PS0190	58	M	4	27	47	57	54	50	65	50	49	50	69	0	0
PS0194	64	F	109	70	145	61	61	65	52	67	67	59	59	0	−8
PS0197	61	F	194	62	70	43	37	42	43	38	47	47	39	0	0
PS0200	62	F	16	41	58	62	61	50	57	51	53	59	54	0	0
PS0223	38	F	35	30	43	61	66	65	65	58	67	59	50	0	0
PS0226	55	M	162	57	73	54	57	55	56	58	58	52	49	0	0
PS0241	51	M	135	34	104	61	55	53	53	67	67	47	49	0	−6
PS0265	45	F	100	36	102	49	49	43	46	56	58	39	47	0	0
PS0288	36	M	156	34	37	51	51	65	57	53	53	50	50	0	0
PS0304	44	M	62	27	31	49	55	46	47	58	62	50	52	0	0
PS0362	56	M	68	92	143	64	66	65	65	67	60	52	56	0	0
PS0396	42	M	50	167	221	60	61	53	55	58	55	56	69	0	−4
PS0426	53	M	34	17	57	62	58	55	55	58	60	49	59	0	0
PS0471	39	F	129	16	48	53	58	57	50	49	58	50	52	2	0
PS0520	50	M	226	91	104	44	37	35	35	54	50	39	39	−2	0
PS0562	44	F	8	14	35	52	59	65	65	60	56	54	54	0	0
PS0639	60	M	70	47	70	66	60	46	45	67	58	56	59	0	0

Spoken object naming/Auditory word repetition/Written object naming/Spoken action naming: maximum score = 75/65/67/69; minimum score = 37/35/38/39; impairment threshold = 62/57/55/62.

### Behavioural data

Each patient was assessed (twice) using the CAT (Swinburn, 2004). For ease of comparison across tasks, task scores are expressed as T-scores, representing each patient’s assessed skill on each task (e.g. describing a picture; reading non-words) relative to a reference population of 113 aphasic patients. The threshold for ‘impairment’ is defined relative to a separate population of 27 neurologically normal control subjects such that performance below threshold would place the patient in the bottom 5% of the normal population (Swinburn, 2004). Lower scores indicate poorer performance.

The CAT yields 34 separate scores, but six of those scores refer to cognitive (non-language) skills and a further six merely summarize other scores, leaving 22 scores that refer to distinct language skills, and a further five, which refer to non-linguistic cognitive skills (Swinburn, 2004). Our focus in this work is on change in scores for the ‘spoken object naming’ task because deficits in this task cause anomia, which is perhaps the most common and frustrating symptom that post-stroke aphasics suffer. But armed with the results of this analysis, we go on to consider change in three other tasks as well: spoken action naming (name the action depicted in a picture), written object naming (write the name of the object in a picture), and auditory word repetition (say the heard word). These analyses serve to help us understand the level of processing at which change might be occurring in the patients’ spoken object naming skills. Details of each of the patients’ scores on these tasks, at the first and second assessment (T1 and T2), are reported in Table 1. Behavioural changes over time were calculated by subtracting the scores at T1 from the scores at T2 for each task, and dividing the result by the months between T1 and T2.

### Structural brain imaging data

Imaging data were collected using sequences described elsewhere (Hope *et al.*, 2015). Data from different scanners were combined after conversion to quantitative probabilistic estimates of grey matter density. Preprocessed with Statistical Parametric Mapping software (SPM, 2012), these images were spatially normalized into Montreal Neurological Institute (MNI) space using a modified version of the unified segmentation algorithm (Ashburner and Friston, 2005) that has been optimized for use in patients with focal brain lesions (Seghier *et al.*, 2008). This creates four types of normalized, segmented images for each structural scan, indexing the probability of: grey matter, white matter, CSF and abnormal tissue. The grey and white matter images each were spatially smoothed with a Gaussian kernel of 8 mm full-width at half-maximum, to minimize any scanner artefacts, then used to calculate within-subject brain changes over time by subtracting the image at the first assessment (T1) from the image at the second assessment (T2). The resulting ‘Brain structure change images’ were divided by the number of months between T1 and T2 to create ‘Brain change rate’ images. This mirrored the procedure we used to calculate rate of object naming change.

The smoothed normalized grey and white matter images were also used in our automated lesion identification tool (Seghier *et al.*, 2008), which indexes the degree of abnormality at each voxel in each patient image (in relation to the same type of images in healthy controls) and combines the grey and white matter outputs to generate a single binary image that shows the presence or absence of a lesion at each voxel. These binary images were used for (i) the lesion overlap maps; (ii) calculating lesion volume; and (iii) excluding patients with lesions smaller than 1 cm^3^ in the left hemisphere or larger than 1 cm^3^ in the right hemisphere.

### In-sample analysis: voxel-based morphometry

This analysis directly tests the hypothesis that the behavioural changes we observed are driven by measurement noise. Measurement noise should be random by definition, and in any case should not be correlated with structural adaptation in the brain. To the extent that such correlations exist between brain change and behaviour change, the implication is that behaviour change cannot be driven entirely by measurement noise—or in other words, that the behavioural change must be ‘real’ or systematic, at least to some extent. In this work, we search for those correlations at the level of individual (2 mm^3^) voxels in the brain.

Voxel-wise associations between rate of brain structure change and rate of behavioural change (for spoken object naming, written object naming and auditory repetition) were measured as partial correlations between brain and behaviour change rate, while accounting for: lesion volume, age at stroke onset, time post-stroke at T1, and task score at T1. All variables were standardized (z-scored) prior to running this analysis; we did this to exclude any artefacts driven by the very different scales on which our variables (including nuisance covariates) were measured. The results were corrected for multiple comparisons using permutation thresholding (Rorden *et al.*, 2009), with 10 000 permutations (*P* < 0.05), and a cluster extent threshold of 30 voxels was applied to the results.

The analysis of grey matter change images included 64 188 right hemisphere voxels with >50% probability of being grey matter, as defined by the standard tissue probability map supplied with the SPM software. We did not expect to detect significant changes in the left hemisphere because (i) the power of the analysis is greatly reduced in voxels where patients have irreparable damage that cannot support structural adaptation; and (ii) our sample included patients with diverse lesion sites that collectively affected the majority of the left hemisphere. Future studies that are able to tightly control for left hemisphere lesion site will be needed to investigate structural adaptation in preserved regions of the left hemisphere.

### Out-of-sample analysis: cross-validation

To attempt to estimate the out-of-sample effect sizes for our brain behaviour associations, we used leave-one-out cross-validation (Ramsden *et al.*, 2012; Price *et al.*, 2013). This is a variant of k-fold cross-validation in which k = *n*, the sample size (28 here). Each fold of the analysis is defined by the selection of a training set and a test set of patients: in leave-one-out cross-validation, the test set includes exactly one patient, and the training set includes all of the other (*n* − 1) patients. Every patient is the test patient in exactly one fold of the analysis. In each fold, we perform the voxel-based morphometry (VBM) analysis using only the training set for that fold, then select the peak voxel (minimum *P*-value) from that analysis. Using linear regression, still focused solely on the training set, we calculate an intercept and slope coefficient for the association between brain change in the peak voxel, and behaviour change in the object naming task. By inverting that regression model, and given the test patient’s rate of brain change in the same voxel, we can predict the test patient’s rate of behaviour change. Each fold of the analysis supplied a predicted behaviour change for a single patient in the object naming task; as in past work (Hope *et al.*, 2013, 2015), we quantified the ‘quality’ of those predictions by correlating predicted and empirical rates of behavioural change, with stronger correlations taken to imply better predictions.

### Are some behavioural changes more predictable than others?

Armed with the predictions from the cross-validation analysis, we asked whether improvements were more predictable than declines or vice versa. We tested this with a Bayesian analysis of variance (Wetzels *et al.*, 2012), with direction of behavioural change as a single factor with three levels (1 = decline; 2 = no change; 3 = improvement), and absolute prediction errors from the cross-validation analysis as the dependent variable. A Bayesian formalism is useful here because it allows us to quantify the evidence both for and against a mediating effect. Where Frequentist equivalents result in one of two outcomes—either evidence for the alternative hypothesis, or no evidence either way—their Bayesian equivalents can also quantify the evidence in favour of the null hypothesis itself.

Bayes factors (BF) > 1 indicate greater support for the alternative hypothesis than for the null hypothesis, and BF < 1 indicate the reverse. Bayes factors of >100 or <1/100 have been called ‘decisive’ evidence for or against the alternative hypothesis, respectively (i.e. the evidence for/against it is more than 100-times stronger than the evidence against/for it) (Jeffreys, 1961). This is the criterion used, in the main text, to assert that improvements and declines in object naming skills are equally predictable: i.e. BF < 1/100. This is really just a stronger way of making the more traditional, Frequentist point that ‘the accuracy (or error) of predicted behavioural changes was not significantly mediated by the observed direction of behavioural change in this task’.

### Functional brain imaging data

The functional MRI data that we report for neurologically normal controls are a novel analysis of the same data reported by Sanjuan and colleagues (2015): a group analysis of 35 neurologically normal participants, engaged in a series of language tasks. That analysis focused on distinguishing the processing of semantically related and unrelated pairs of objects in non-spoken semantic decisions, whereas ours considers whether the right hemisphere regions showing structural plasticity are involved in naming and semantic tasks. The paradigm included five separate tasks: (i) making semantic association decisions on two visually presented pictures of objects (with finger press response); (ii) naming two visually presented pictures of objects; (iii) making semantic association decisions on pairs of heard object names (again with finger press responses); (iv) describing the scene depicted in a picture; and (v) naming the verb that describes an action depicted in a picture. We only report the comparison of object naming to visual semantic decisions (i > ii) because these conditions were matched for sensory inputs and semantic content. Although we focus on regions of interest, we corrected for multiple comparisons across the entire brain using family-wise error correction—derived from random field theory, as implemented in the SPM software (SPM, 2012)—because we did not have an *a priori* hypothesis as to the nature of the effect.

## Results

### Lesion data, object naming skills, and speech and language therapy

**Figure 1:**
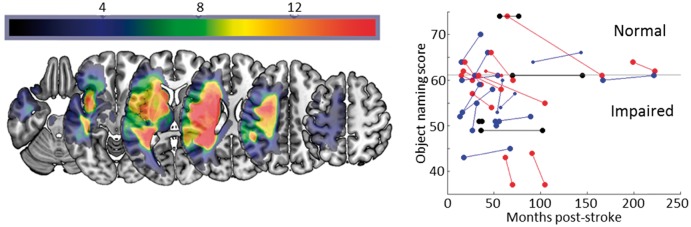
**Lesion and behavioural data.***Left*: Axial slices of a lesion frequency image for the patients. *Right*: Scores in the object naming task at first and second assessment. Blue = improvement; black = no change; red = decline.

Figure 1 illustrates the distribution of the patients’ lesions and the changes observed in the object naming task between first (T1) and second (T2) assessments. We observed improvement in 13/28 patients and decline in 11/28, though there was no main effect of change across the group (*t* = 0.28, *P* = 0.78). We had responses from 17/28 patients concerning their experiences of speech and language therapy, though the form of that therapy was very variable across the group. Just six of those patients reported having any formal therapy at all between their first and second assessments: object naming skills improved in three of those six during the same period, declined in two, and were stable in one patient. The most rapidly improving patient reported an end to formal therapy nearly 2 years prior to the date of their first assessment here. Given the missing data on this point for 11/28 patients, we cannot draw strong conclusions, but given the data we do have, speech and language therapy does not appear to be driving the changes we observed. Taken in isolation, these results are consistent with the common view that chronic aphasics’ language skills are fundamentally stable, with the implication that the individual changes are driven by measurement noise.

### Associating behavioural change with structural adaptation in the brain

**Figure 2:**
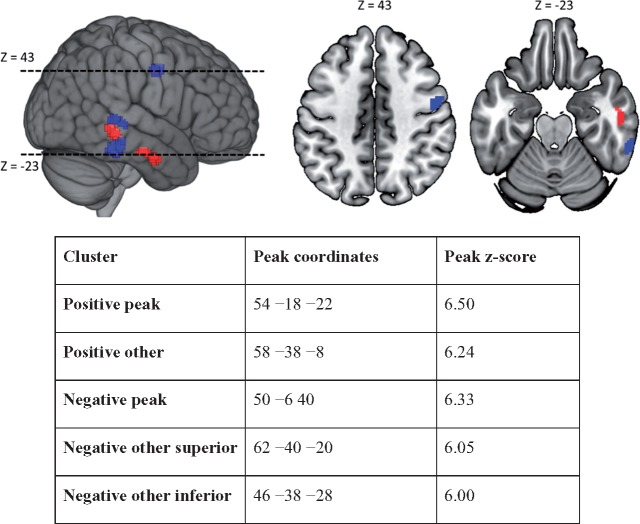
**VBM results.** Five clusters of voxels survive a whole brain correction for multiple comparisons: two positive clusters (in red, associating increased grey matter with increased task score) on the right middle temporal gyrus, with the more posterior of the two bracketed by two negative clusters (in blue associating decreased grey matter with increased task score), and a further negative cluster on the right precentral gyrus (premotor area 6). Peak positive and negative clusters are displayed in axial slices (peak negative at Z = 43 mm and peak positive at Z = −23 mm). The table displays peak coordinates for all five clusters, together with the Z-score at that peak.

We searched for those associations using VBM, running voxel-wise partial correlation analyses between the rate of behavioural change and the rate of brain structural change, while controlling for lesion volume, age at stroke onset, time post-stroke at T1, and object naming score at T1. Five clusters of voxels survived a correction for multiple comparisons across all included voxels; two with positive and three with negative correlations between rates of brain structural adaptation and rates of behavioural change. The ‘positive’ clusters (i.e. associating increasing grey matter with increasing task score and vice versa) were both on the right middle temporal gyrus, with the peak voxel in the more anterior cluster referred to as the ‘peak positive cluster’ in what follows (Fig. 2). Two of the ‘negative clusters’ (i.e. associating increasing grey matter with decreasing task score and vice versa) were in similar areas on the right middle and inferior temporal gyri, respectively, but the ‘peak negative cluster’ was on the right precentral gyrus, principally in premotor area 6 (Fig. 2). That behavioural change in this task should be so strongly correlated with brain change, is not consistent with the view that the either are driven by measurement noise.

### Predicting behavioural change given structural adaptation in the brain

VBM can be sensitive to outliers (Nocchi *et al.*, 2008), which may inflate in-sample effect sizes. As further validation of the in-sample effects found so far, we used cross-validation to estimate the extent to which we could predict new patients’ rates of behavioural change, given their rates of brain change (Ramsden *et al.*, 2012; Price *et al.*, 2013). Detailed in the ‘Materials and methods’ section, this process involved making predictions for each patient’s behaviour change based on their brain change in a peak voxel identified via VBM analyses of the other patients. To confirm the significance of both directions of in-sample effect, we report analyses restricted to positive and negative correlations, respectively.

**Figure 3:**
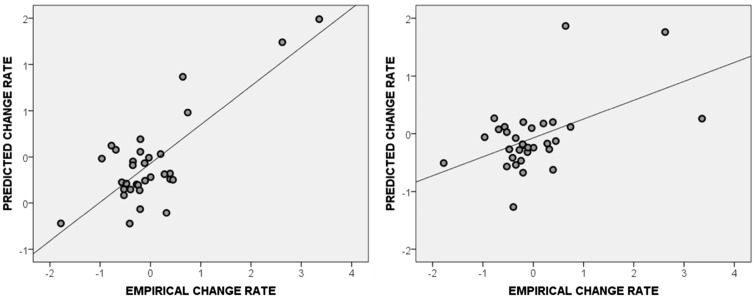
**Cross-validation results.** Predicted versus actual rates of behavioural change in the (*left*) positive-only and (*right*) negative-only analyses. In each case, the predictions are significantly correlated with the empirical changes.

Every fold of the positive-only cross-validation analysis (i.e. accepting only voxels with a positive correlation between increasing grey matter and improving scores) selected a voxel inside the peak positive cluster, and the resultant predictions were strongly correlated with the patients’ empirical rates of behavioural change (r = 0.88, *P* < 0.001) (Fig. 3). The negative-only cross-validation analysis was less consistent, because voxels inside the peak negative cluster were only selected in 23/28 folds of the analysis, but the resultant predictions were still significantly related to the patients’ real rates of change (r = 0.46, *P* = 0.015). Strong predictions were still generated when each analysis was repeated after excluding Patients PS0171 and PS0304, whose skills appeared to improve much more quickly than the rest (positive-only: r = 0.54, *P* = 0.004; negative-only: r = 0.56, *P* = 0.003). In this sample, individual participants’ rates of behavioural change were both correlated with and predictable given rates of structural adaptation in the right hemisphere of the brain.

### Declines in object naming skill are just as systematic as improvements

Our in-sample (VBM) and out-of-sample (cross-validation) analyses demonstrate that the patients’ behavioural changes are systematic and predictable, at least to some extent. Previous studies, which highlight the potential for change in chronic aphasia, overwhelmingly emphasize recovery as the counterpoint to presumed stability (Moss and Nicholas, 2006; Plowman *et al.*, 2012; Teasell *et al.*, 2012). In that context, the declines that we observed—in the absence of intervening stroke events, or significant comorbid conditions (see ‘Materials and methods’ section)—might be more surprising than the improvements. This naturally begs the question of whether our previous results are being driven by systematic improvements alone.

To test this, we used a Bayesian ANOVA (Wetzels *et al.*, 2012), with direction of behavioural change as a single factor with three levels (1 = decline, 2 = no change, and 3 = improvement), and absolute prediction errors from the cross-validation analysis as the dependent variable: i.e. the differences between predicted and empirical rates of behavioural change. If the effects reported so far are driven by systematic improvements alone, those improvements should be more predictable than the declines: the direction of change should mediate the predictability of change.

Here, the evidence against this effect was 112 times stronger than that for it (i.e. Bayes factor = 0.0090) in the positive-only analysis, and 111 times stronger than that for it (Bayes factor = 0.0089) in the negative-only analysis. The implication is that we could predict improvements and declines equally well in both analyses—given growth or atrophy in the same regions—suggesting that both directions of change were equally systematic.

### Structural adaptation occurs within word-retrieval/articulation areas

Next, we asked whether the areas showing structural plasticity with changes in object naming ability are normally involved in object naming in the undamaged brain. This involved functional MRI in a separate sample of 35 neurologically normal controls, as described previously, with a focus on two tasks: naming two visually presented objects, and making semantic association decisions on visually presented objectes with a finger press response.

Both the peak positive and peak negative clusters were significantly activated by object naming relative to the semantic decision with finger press task (positive cluster peak voxel at [48, −6, −30], Z = 5.24, *P* < 0.05; negative cluster peak voxel at [48, −9, 36], Z > 8, *P* < 0.05; both corrected for multiple comparisons across the whole brain). Moreover, there was no significant activation relative to rest in either cluster in the semantic decision (finger press) task, even at a permissive threshold (*P* < 0.05, uncorrected). The contrast of object naming with visual semantic matching controls for visual processing, object recognition and semantic associations; we conclude that voxels in these clusters serve either word-retrieval or articulation functions in the undamaged brain, consistent with their apparent relevance to the ‘word-finding difficulties’ (anomia) that our VBM analysis was designed to probe.

### Word-finding or articulation? Evidence from other tasks

The functional MRI results suggest that the changes in our patients’ spoken object naming skills might reflect changes in either their word-finding or their articulation skills. To try to distinguish between these two accounts, we first searched for other CAT tasks where score changes were positively correlated with the changes we observed in the spoken object naming task. Controlling for the same nuisance covariates and for multiple comparisons as in the previous analyses, we found changes in the spoken object naming scores were significantly correlated with changes in: (i) written object naming scores (r = 0.71, *P* < 0.001); and (ii) spoken action naming scores (r = 0.67, *P* < 0.001) (Table 1). In contrast, the correlation between spoken object naming and auditory repetition scores was not significant (r = 0.04, *P* = 0.83). This set of results cannot explain the change in spoken object naming results in terms of a change in articulation abilities as this would have resulted in spoken object naming scores being more strongly correlated with auditory repetition (high demands on overt articulation) than written object naming (no demands on overt articulation). The results are, however, consistent with the change in spoken object naming scores being a consequence of changes in word finding abilities (lower during auditory repetition than spoken object naming, spoken action naming and written object naming). This was formally demonstrated by showing that spoken object naming was significantly more correlated with written object naming than auditory repetition (z = 3.00, *P* = 0.003).

**Figure 4:**
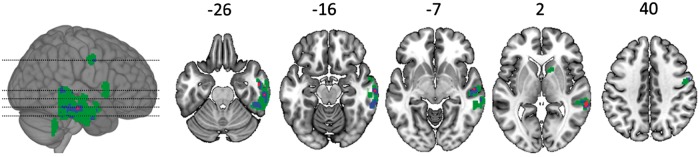
**Brain-behaviour associations across three language tasks**. A frequency image of voxels where structural adaptation was significantly associated with behavioural change in three tasks where behavioural change was significantly correlated across tasks: (i) object naming; (ii) action naming; and (iii) written object naming. Green voxels were significant in just one task (after correcting for multiple comparisons), blue voxels were significant in two tasks, and red voxels were significant in three tasks.

The brain changes provide further support that the change in spoken object naming reflected changes in word finding abilities. Structural adaptation in the right middle temporal sulcus correlated with changes in spoken object naming, written object naming and spoken action naming (Fig. 4) but not auditory repetition. A comparison of these effects confirmed that structural adaptation in the regions identified by spoken object naming was significantly more correlated (after correction for multiple comparisons) with changes in written object naming than changes in auditory repetition: Z = 4.44, *P* < 0.001 at [+50, −10, −30]; and Z = 3.56, *P* = 0.002 at [50, −8, 38]. This is the expected result if the improvements and declines that we observe in the object naming task reflect variation at the level of word-finding processes in the brain.

### No simple predictors of improvement or decline

Finally, we asked whether there were any obvious predictors of improvement or decline in the patient data available at T1. There were no significant differences between the ‘improvers’ and ‘decliners’ in terms of age at stroke onset (t = 0.86, df = 22, *P* = 0.40), time post-stroke at T1 (t = 0.48, *P* = 0.64) or T2 (t = 0.61, *P* = 0.55), or lesion volume (t = 0.40, *P* = 0.69), and also no sex differences (Wilcoxon signed ranks test: z = 0.53, *P* = 0.53). The left homologue of our peak positive cluster (in the left anterior temporal lobe) was intact in all patients, and there was no consistent effect of the damage that 9/28 patients had to the left homologue of the peak negative cluster (in the left premotor cortex: t = 1.64, df = 26, *P* = 0.11). A VBM analysis associating rates of behavioural change with grey matter at T1 alone (controlling for our nuisance covariates, as in the main analysis) showed no consistent effects anywhere in the brain. And there was also no consistent relationship between mean grey matter signal intensity at T1, within our peak VBM clusters, and rates of subsequent behavioural change (peak positive cluster: r = −0.05, *P* = 0.80; peak negative cluster: r = 0.18, *P* = 0.37). In other words, we found no simple predictors of the direction or extent of behavioural change, in the patient data available at T1.

## Discussion

At the group level, our sample of chronic aphasics’ object naming skills appeared to be stable, consistent with the prevailing view that their recovery has plateaued. There was also no significant correlation between the patients’ task scores and the times post-stroke at which they were assessed (r = 0.14, *P* = 0.45); this is another criterion that was recently used to support the impression that a plateau has been reached (Xing *et al.*, 2016). Nevertheless, we found that this apparent stability belies a dichotomy of improving and declining language skills at the level of individual patients. The changes that we observed were systematically associated with, and predictable given, structural adaptation in the (intact) right hemisphere of the brain, and declines were just as systematic and predictable as improvements. These results are not consistent with the view that the changes we observed are artefacts of measurement noise. On the contrary, they imply that real change continues for years.

Further support for this contention flows from our analyses of correlated change in other task scores. Changes in the (spoken) object naming scores were significantly, positively correlated with change in two other word-finding tasks (written object naming and action naming), and change in each of those tasks was also independently and significantly associated with structural adaptation in brain regions which overlap with those found for spoken object naming (Fig. 4). In other words, we observed similar behavioural changes, associated with structural adaptation in similar brain regions, in three similar tasks. As there is no reason to expect that measurement noise should group these tasks together, we suggest that the grouping itself is further evidence that these behavioural changes are not driven entirely by measurement noise.

Using functional MRI in a separate sample of neurologically normal controls, we confirmed that the ‘peak VBM regions’, where structural adaptation was most strongly associated with changing object naming scores, are normally involved in object naming, and significantly more involved in naming than in visual semantic decisions. This finding is broadly consistent with the emerging emphasis on bilaterality in models of anterior temporal lobe function (Rice *et al.*, 2015). The contrast with the visual semantic decision task controls for visual processing, object recognition and semantic associations, so we concluded that the activity in these regions reflects either word-retrieval or articulatory processing.

We refined that interpretation further by reference to score changes in other tasks: a written object naming task, which requires word-retrieval but not overt articulation, and an auditory word repetition task, which requires articulation but not (or less) word retrieval. Change in written object naming was significantly correlated both with change in (spoken) object naming and with structural adaptation in our peak VBM clusters, and both of these effects were significantly stronger than those observed for auditory word repetition. These results suggest that change in the patients’ spoken object naming skills is driven by change in their word-finding skills.

One immediate consequence of this result is that doubt is cast on the conventional measures of ‘measurement noise’ for the CAT. The manual that describes the CAT also provides a formal test-retest reliability analysis, which aggregates observations from two samples of stroke survivors: one that is similar to our own (21 chronic patients, tested at 5–15 week intervals), and another that is still more acute (48 patients, tested at 6 and 12 months post-stroke). The authors treat all observed score changes as ‘measurement noise’, assuming that all of these patients’ language skills are fundamentally stable: i.e. making precisely the assumption that our results undermine. If our results are right, the implication is that the CAT authors’ own calculation of measurement noise could be confounded by real change, which in turn suggests that their measurement of that noise might be inflated.

Our longitudinal results also complement and extend those of a recent, cross-sectional study, which uses VBM to correlate structural differences in the right hemisphere with language outcomes after left-hemisphere stroke (Xing *et al.*, 2016). Those authors not only found significant associations between relative grey matter and relative language outcomes, but also differences in the same regions between the aphasics and a separate sample of neurologically normal controls. Both results could reflect premorbid differences in brain structure, but the latter (the difference relative to controls) encourages the view that post-stroke adaptation, or regional hypertrophy, plays some role (Xing *et al.*, 2016)—albeit that the timing of that adaptation is left somewhat vague because it could have occurred during the acute phase post-stroke. Our results demonstrate—for the first time, as far as we know—that this adaptation does in fact occur, and also that it occurs well into the chronic phase. We interpret this hypertrophy as the footprint of sustained functional enhancement in the same regions (Maguire *et al.*, 2000). As the enhancement occurs in regions that are normally activated when people name objects, the implication is that it reflects some sort of compensation—working residual parts of the object-naming network harder to make up for damage elsewhere.

Another surprising feature of our results is that contrasting patterns of structural change—which might reflect different compensatory ‘strategies’—are associated with contrasting behavioural effects. Functional enhancement in the right anterior temporal lobe (the peak positive cluster) was associated with improved language skills, but functional enhancement in right premotor cortex (the peak negative cluster) was associated with declining skills. Rates of structural adaptation in these two regions were strongly anti-correlated across the group (r = −0.95, *P* < 0.001), suggesting a consistent division between the patients who adapted well and those who adapted poorly. The observation that neighbouring brain regions in the right middle and inferior temporal gyrus also showed opposing types of structural changes (increases or decreases in grey matter as object naming improved) is intriguing. A similar finding was also observed in taxi drivers who had learnt ‘the knowledge’ with increased structural volumes in posterior hippocampus and decreased volumes in anterior hippocampus. The authors of that study (Maguire *et al.*, 2000) argue that although the differential changes in posterior and anterior hippocampus may represent two separate processes, the most parsimonious explanation is one of a more general reorganization of function with changes in one region inevitably affecting other neighbouring regions.

There was no simple predictor of behavioural improvement or decline, at least that we could find, in the patient data available at T1. The distinction between improvers and decliners might reflect some more subtle (e.g. more multivariate) distinction between the patients, or it might reflect variable patterns of everyday language use or attempted use (i.e. ‘use-dependent plasticity’), or some combination of those and other factors. Indeed, while the data we have concerning the patients’ experiences of speech and language therapy cannot explain their behavioural trajectories, that data are missing in 11/28 patients, and do not in any case address those less formal aspects of their environment (such as the efforts of an attentive carer) which might be driving the change. Our results demonstrate that improvements and declines are happening, but as yet, we cannot confidently explain why those changes occur.

We also cannot confirm what the functional or day-to-day significance of these behavioural changes might be. By design, we have decoupled the issue of real versus artefactual change from the magnitude of that change; we contend that behavioural change is ‘real’ if it is associated with (and predictable given) brain change, irrespective of its magnitude. But even without that distinction, the relationship between our impairment-based measures of language skill, and the patients’ day-to-day experiences of language use, might be complex. For some, learning a single new word might transform their normal conversation, while others might feel less practical benefit even with many more words. Further work is needed to bridge the gap between the impairment-based measures, like ours, which dominate the academic study of aphasia, and the experiences that patients have day-to-day.

Finally, it seems natural to ask whether interventions might be used to encourage patients away from the apparently maladaptive compensatory strategy that we have observed here, and toward the more adaptive strategy. One natural candidate for such an intervention would be neural stimulation, for example using low frequency repetitive transcranial magnetic stimulation (rTMS) to suppress the region where our peak negative cluster occurred (Fig. 2). There is already promising evidence that this kind of intervention can drive improvements in aphasics’ language skills when applied to the right inferior frontal gyrus (i.e. in and around the right homologue of Broca’s area, typically associated with speech production) (Ren *et al.*, 2014), but as far as we know, the few prior studies that have considered right premotor cortex for suppression have been focused exclusively on motor impairments (O’Shea *et al.*, 2007; Kaski *et al.*, 2014). Nevertheless, there is evidence that stimulation applied in other motor regions (specifically, left primary motor cortex) can drive improvements in aphasia (Meinzer *et al.*, 2016); given the results reported here, we would predict that suppression of the right premotor cortex might encourage recovery or arrest decline in object naming skills even years after left hemisphere stroke. The same hope extends to excitatory stimulation (e.g. using high frequency rTMS or anodal transcranial direct current stimulation) of the right anterior temporal region where our peak positive cluster occurred (Fig. 2), and also in principle to the other more posterior clusters that we found. However, these more posterior clusters may be too close together in practice to permit the selective excitation and suppression that our results suggest would be required.

Whether or not these predictions bear fruit, we hope that this result will encourage further scrutiny of the trajectories that chronic aphasics’ language skills are taking, and further research into the factors that mediate those trajectories. There is an emerging paradox here, with an increasing weight of evidence that chronic aphasics’ language skills can and do change, with or without intervention, contrasted with the still steadfast pessimism in the clinical community and a consequent lack of resources to treat these patients beyond the acute phase (Teasell *et al.*, 2012). For the sake of those patients with chronic aphasia who might otherwise believe they can never recover, we hope that our results add weight to the case for change.

## Abbreviations

CAT: Comprehensive Aphasia Test
VBM: voxel-based morphometry
